# The “Mange,” Communicated to Three Persons by a Pig

**Published:** 1850-12

**Authors:** 


*The "Mange," communicated to three persons by a Pig.* Reported in a letter to the Editor. By H. R. CASEY, M. D., of Columbia county, Ga.

I will give you the particulars of a conversation held a few days since with a gentleman of this county, and if the deduction I have drawn from the facts as reported is correct, we have presented to us (so far at least as my observation extends) a new disease of the cutaneous system—one hitherto undescribed by dermatologists.

Mr. S. asked me "if I had ever known a man to have the mange?" to which I gave a negative reply: having always understood that it was a disease peculiar to the quadruped—He then asked me "if I thought it possible for a man to catch it from a hog?" I replied, that there are a great many things regarded as impossible, which are not found to be so when subjected to the test—and that this might be one of the cases. He then proceeded to give me the following particulars:

He states that about the first of May last, having a pig badly diseased with the mange, and being desirous to cure him, he had some soap and water got and went to work on him with his hands—and that after giving him a good washing, he stripped him almost of his entire *external* with his nails. That he was entirely well at this time; but that in about three hours thereafter, he felt an itching on his hands and wrists, and an eruption which commenced spreading upwards; that about the same time, his ankles began to itch him and the eruption there made its appearance, which also spread upwards and met the eruption from above at the half-way house—the umbilicus; that it reached its height in about two weeks; that the eruption was characterized by great heat and intolerable itching, composed of small vesicles, which, though not confluent, stood close together over his entire tegumentary tissue. Thus was he at the time of his commencement with the ablution—a sound and healthy man—but in a very short time thereafter, he was transformed into a Lazarus. He thought he had contracted his disease from the pig, and went to work to cure himself, using first the soap and water. This not benefiting him, he was bled and took salts. This failing, he tried *pot-liquor*—then the grease from fried bacon—then a solution of blue-stone. He does not think that any of the means used had any control whatever over the disease, but that it seemed to pursue its course, knowing no conqueror, until it finally wore itself out, in about five weeks.

Now, from the above narrative, I can but infer that the disease in question was one identical with the mange, and that it was communicated from the quadruped to the man. And I am further strengthened in this view of the case, from the fact—that a female and the negro boy who held the pig while being subjected to treatment, became in like manner affected. The view I have taken of this case, I know to be in direct conflict with the long-established dogmas of the veterinary school, but I think I am sustained in my position from the facts of the case—and "facts are stubborn things."

By reference to the "*History of the Horse,*" I find the following language. The author, in speaking of the contagiousness of the mange, goes on to say —"If the same brush or curry-comb be used on all the horses, the propagation of mange is assured; and horses feeding in the same pasture with mangy ones, rarely escape, from the propensity they have to nibble one another. Mange in cattle has been propagated to the horse—and from the horse to cattle—but there is no authenticated instance of the same disease being communicated from the dog to the horse. There is as much difference in the character and eruption of mange in the horse and dog, as between either of them and the itch in the human subject; and the itch has never been communicated to the quadruped, *nor the mange of the quadruped to the human being.*"

My only reply to the above quotation, is the presentation of the case related; and if I am not sustained in my corollary from the facts of the case, this article will go for nothing. I pretend to no familiarity with cutaneous diseases; but if I were called upon to classify the mange, I should locate it in the group *dermatoses scabienses* of Wilson, not only from the pathology, but also from the therapeia of the disease; for I find sulphur the anchor of safety to the veterinary surgeon. Nor do I think there is any thing very strange in all this; and the only reason why we have never before had the mange communicated to man arises simply, I think, from the fact, that in all probability more caution has hitherto been experienced than was in the case before us. We have examples of other diseases occurring in the human subject, the result of propagation from the lower order of animals. In the Revue Medicale, of July, 1845, we have detailed a case of an officer who took the glanders and farcy from a horse, and in which experiments were made by M. Andouard, to test the contagiousness of the human fluid introduced into other animals—the results of which experiments went to prove that the disease was not only communicable to man from the horse, but that the disease was again transmissible from the human subject to the quadruped. In the Southern Medical and Surgical Journal, Nov., 1847, we have a case of Glanders in the human subject, derived from the horse, reported as occurring in your own city. Other diseases might be mentioned occurring in the great paragon of animals, communicated from the lower order; but I have already spun out this article to a greater length than was designed at its commencement, and will conclude by merely advising those persons who may have to treat the mange in stock, to touch it lightly, and never make a curry-comb of their hands; to which injunction I know my friend F. will say amen.—*Southern Med. Jour.*

